# Expression of the IL-7 Receptor Alpha-Chain Is Down Regulated on the Surface of CD4 T-Cells by the HIV-1 Tat Protein

**DOI:** 10.1371/journal.pone.0111193

**Published:** 2014-10-21

**Authors:** Denny McLaughlin, Elliott Faller, Scott Sugden, Paul MacPherson

**Affiliations:** 1 Department of Biochemistry, Microbiology and Immunology, Faculty of Medicine, University of Ottawa, Ottawa, Ontario, Canada; 2 Department of Medicine, Faculty of Medicine, University of Ottawa, Ottawa, Ontario, Canada; 3 Division of Infectious Diseases, Ottawa Hospital General Campus, Ottawa, Ontario, Canada; 4 Ottawa Hospital Research Institute, Ottawa, Ontario, Canada; INRS - Institut Armand Frappier, Canada

## Abstract

HIV infection elicits defects in CD4 T-cell homeostasis in both a quantitative and qualitative manner. Interleukin-7 (IL-7) is essential to T-cell homeostasis and several groups have shown reduced levels of the IL-7 receptor alpha-chain (CD127) on both CD4 and CD8 T-cells in viremic HIV+ patients. We have shown previously that soluble HIV Tat protein specifically down regulates cell surface expression of CD127 on human CD8 T-cells in a paracrine fashion. The effects of Tat on CD127 expression in CD4 T-cells has yet to be described. To explore this effect, CD4 T-cells were isolated from healthy individuals and expression levels of CD127 were examined on cells incubated in media alone or treated with Tat protein. We show here that, similar to CD8 T-cells, the HIV-1 Tat protein specifically down regulates CD127 on primary human CD4 T-cells and directs the receptor to the proteasome for degradation. Down regulation of CD127 in response to Tat was seen on both memory and naive CD4 T-cell subsets and was blocked using either heparin or anti-Tat antibodies. Tat did not induce apoptosis in cultured primary CD4 T-cells over 72 hours as determined by Annexin V and PI staining. Pre-incubation of CD4 T-cells with HIV-1 Tat protein did however reduce the ability of IL-7 to up regulate Bcl-2 expression. Similar to exogenous Tat, endogenously expressed HIV Tat protein also suppressed CD127 expression on primary CD4 T-cells. In view of the important role IL-7 plays in lymphocyte proliferation, homeostasis and survival, down regulation of CD127 by Tat likely plays a central role in immune dysregulation and CD4 T-cell decline. Understanding this effect could lead to new approaches to mitigate the CD4 T-cell loss evident in HIV infection.

## Introduction

CD4 T-cell depletion is a hallmark of HIV disease progression. The exact mechanisms by which HIV causes CD4 T-cell loss, however, have yet to be fully elucidated. While direct cytopathic effects of HIV and activation of HIV-specific natural killer cells and cytotoxic T-cells are two important means by which HIV-infected CD4 T-cells may be eliminated, these mechanisms likely explain only a portion of the loss given less than 0.2% of the peripheral CD4 T-cell population is productively infected [1], [2], [3]. Chronic immune activation with T-cell exhaustion [4], impaired T-cell production [5], and increased CD4 T-cell susceptibility to apoptosis have also been suggested to account for the dramatic decline in CD4 T-cells in infected individuals [6]. Of note, quiescent CD4 T-cells are particularly susceptible to death by caspase-1 mediated pyroptosis induced by accumulation of incomplete HIV reverse transcripts resulting from abortive infection [7], [8].

Interleukin (IL)-7 is pivotal to T-cell survival and homeostasis and plays an important role in maintaining constant numbers of naïve and memory CD4 and CD8 T-lymphocytes in the peripheral circulation [9]. IL-7 promotes T-cell proliferation by stimulating entry into the cell cycle[10], [11], [12], [13] and enhances T-cell survival by up regulating the anti-apoptotic factors Bcl-2 and Bcl-xL [14] while inhibiting the pro-apoptotic factors Bad and Bax [15]. IL-7 signals through the IL-7 receptor, a heterodimeric complex comprised of a unique α-chain (CD127) and the common γ-chain (CD132) that is shared with the receptors for IL-2, -4, -9, -15, and -21. CD127 is highly expressed on naïve and memory CD4 and CD8 T-cells [16], [17]. In the absence of IL-7 signaling there is a substantial depletion of T-cells from the peripheral circulation [18].

We and others have shown decreased expression of the IL-7R α-chain (CD127) on CD4 and CD8 T-cells in HIV-infected individuals [19], [20], [21], [22], [23], [24], [25], [26]. Loss of this receptor subunit has been shown to correlate with CD4 T-cell decline [24] and disease progression in HIV-infected patients [22], [24], [26], [27]. Notably, reduced CD127 expression on the surface of CD4 T-cells in viremic HIV+ patients results in decreased responsiveness to the anti-apoptotic effects of IL-7 [28] likely contributing to CD4 T-cell apoptosis and depletion. Together, these data suggest suppression of CD127 expression on CD4 T-cells during HIV infection results in homeostatic imbalance and contributes to the loss of circulating CD4 T-cells.

We have previously shown down regulation of CD127 on the surface of CD8 T-cells is mediated at least in part by soluble HIV Tat protein [27]. Tat, a small 14 kdal HIV regulatory polypeptide, acts in a paracrine fashion to alter the function of neighboring cells [29], [30]. This small protein is secreted by HIV-infected cells and can be found in the media during in vitro infection [31], [32] as well as in the serum of HIV-infected patients [33]. Secreted Tat is rapidly internalized by a variety of cell types [32], [34], [35] by binding via its arginine-rich basic domain to heparan sulfate proteoglycans on the cell surface [35], [36], [37] and is then internalized by endocytosis [35], [38], [39], [40]. When added to purified CD8 T-cells isolated from healthy HIV-negative donors, soluble Tat protein induces a significant reduction in CD127 surface expression compared to cells maintained in medium alone [27]. We have recently demonstrated soluble Tat protein enters CD8 T-cells by endocytosis and exits late endosomes through a process dependent on the usual acidification of these vesicles [41]. Once inside the cytoplasm, Tat translocates to the inner leaflet of the plasma membrane where it binds directly to the cytoplasmic tail of CD127 [41]. This interaction with Tat induces receptor aggregation or capping and removal from the cell surface through a process dependent on microtubules [41]. Finally, Tat directs CD127 to the proteasome for degradation. Through this mechanism, Tat decreases the half-life of CD127 in CD8 T-cells by more than three-fold [41]. Importantly, not only does Tat down regulate CD127 on CD8 T-cells, but this down regulation prevents accumulation of intracellular perforin in response to IL-7 [27]. The significant role the HIV Tat protein plays in removing CD127 from the surface of CD8 T-cells lead us and others to ask if it had the same effect on CD4 T-cells. By decreasing CD127 expression on the surface of CD4 T-cells, Tat could disrupt IL-7 signaling leading to reduced Bcl-2 expression and increased CD4 T-cell apoptosis and depletion. As hypothesized, we found that similar to CD8-T cells, CD127 is down regulated from the surface of CD4 T-cells by the HIV-1 Tat protein and degraded by the proteasome. This down regulation was dose dependant and resulted in a significant reduction in IL-7 induced Bcl-2 production. In addition to extracellular Tat having this effect, we also demonstrate that Tat produced endogenously also down regulates CD127 expression on CD4 T-cells.

## Materials and Methods

### Ethics statement

This work was reviewed and approved by the Ottawa Hospital Research Ethics Board and informed written consent was obtained from all participants. No children were used in this study. Written consent was obtained from all participants after reading a 5 page document describing phlebotomy, study outline, and research goals.

### Isolation and culture of Primary CD8+ and CD4+ T-Cells

Peripheral blood mononuclear cells (PBMC) were isolated by Ficoll-Paque density centrifugation from blood of healthy adult volunteers with no known risk factors for HIV infection. CD8+ T-cells were positively selected as previously described using the MACS Microbead CD8+ Cell AutoMACS Isolation System (Miltenyi Biotec, Bergisch Gladbach, Germany) according to the manufacturer's directions [27]. Purified CD8 T-cells were resuspended at 10^6^ cells/ml in RPMI 1640 (Hyclone, Logan, Utah) medium supplemented with 20% fetal calf serum (FCS; Cansera, Rexdale, Ont. Canada) plus 100 units/ml penicillin, 100 µg/ml streptomycin and 0.2 M L-glutamine (RPMI-20). The negative cell fraction was also suspended in RPMI-20 and incubated in a polystyrene tissue culture flask (BD Falcon) at 37°C for 75 minutes to allow CD4+ monocytes to adhere to the surface. The medium containing nonadhered lymphocytes was then decanted into a new tube and CD4 T-cells were isolated in the same manner as for the CD8 T-cells using anti-CD4 antibody conjugated ferromagnetic beads (Miltenyi Biotec). Purified CD4 T-cells were resuspended in RPMI-20 at 10^6^ cells/ml. In order to confirm purity the cells were analyzed for CD4 and CD14 expression and were consistently >97% pure CD4 T-cells.

The isolated T-cells were maintained in medium alone or suspended in medium containing purified HIV-1 Tat protein (Advanced Bioscience Laboratories, Inc. Kensington, MD) or recombinant human IL-7 (Invitrogen Biosource. Carlsbad, CA) as specified. All cultures were incubated at 37°C in 5% CO_2_.

To block Tat's effect, purified Tat protein was pre-incubated with heparin (Organon; 10 µl/ml to give final concentration of 100 USP units/ml) or rabbit anti-Tat polyclonal antibodies (Diatheva; ANT000) at a final concentration 140 µg/ml. Inhibitors were purchased from Sigma Aldrich, St Louis, MA. Cells were pre-incubated with and without proteasome inhibitors (5 µM MG132, 10 µM Lactacystin), lysosome inhibitors (10 µM Leupeptin, 10 µg/ml Pepstatin) and 100 µM cyclohexamide for 1 hour prior to incubation with and without 10 µg/ml Tat protein.

### Flow cytometry

Cells were incubated with the indicated flourochrome-labeled antibodies for 20 minutes in the dark at room temperature and analyzed immediately using a Coulter Epics ALTRA flow cytometer (Fullerton, CA). Live cells were gated on the basis of side and forward scatter and at least 10,000 events were recorded for each sample. The following antibodies were obtained from Immunotech Beckman Coulter (Marseille, France): anti-CD127-phycoerythrin (PE) (R34.34), anti-CD3-fluorescein isothiocyanate (FITC) (UCHT1), anti-CD4-FITC (13B.2), anti-CD8-phycoerythrin-Cy5 (PC5) (B9.11), anti-CD14-FITC (RM052), anti-CD25-PC5 (B1.49.9), anti-CD28-PC5 (CD28.2), anti-CD45RA-FITC (ALB11), anti-CD45RO-FITC (UCHL1), anti-CD56-PC5 (N901NHK-1), and anti-CD62L-FITC (DREG56). Anti-CD132-PE (555900) was obtained from BD Biosciences (Mississauga, Ontario, Canada). All antibodies were used at saturating concentrations and the corresponding isotype controls were included.

### Cell viability assays

Purified CD4 T-cells were incubated in medium alone or with Tat protein (10 µg/ml) in a humidified incubator at 37°C with 5% CO_2_. Camptothecin (10 µM, Sigma-Aldrich) was used as a positive control for apoptosis. At 24 and 72 hours, cells were stained with Annexin V-FITC and propidium iodide (PI) using the Apoptosis Detection Kit I (BD Biosciences) according to the manufacturer's directions.

### Intracellular staining for BCL-2

Purified CD4 T-cells were pre-incubated at a concentration of 1×10^6^ cells/ml either in RPMI-20 alone or with Tat protein (10 µg/ml) for 48 hours. Recombinant human IL-7 (10 ng/ml) was then added and the cells were incubated at 37°C for an additional 72 hours. Following treatment, the cells were fixed in 2% paraformaldehyde in phosphate buffered saline (PBS) (PFA) at room temperature for 10 minutes. The samples were then washed twice with PBS containing 1% bovine serum albumin (BSA) and permeabilized by resuspending in cold methanol for 10 minutes at 4°C. The cells were again washed twice as above and resuspended in 100 µl PBS with 1% BSA. The cells were then stained with FITC-conjugated mouse anti-human Bcl-2 monoclonal antibodies (Bcl-2/100; BD Biosciences) or FITC labeled mouse IgG, κ isotype control (MOPC-21; BD-Biosciences) for 45 minutes at room temperature in the dark. The cells were then washed as above, resuspended in PFA and immediately analyzed by flow cytometry.

### Primary CD4 T Cell Nucleofection

Primary CD4 T-cells were isolated as described above and cultured overnight in RPMI-20. The next day cells were stimulated with anti-CD3/anti-CD28 antibody conjugated ferromagnetic beads using the T-cell activation/expansion kit from Miltenyi Biotec (Bergisch Gladbach, Germany). After 24 hours, beads were removed by magnetic separation and CD4 T-cells were nucleofected using the human T-cell nucleofector kit *(*Lonza, Basel, Switzerland*)*. Briefly, 5×10^6^ CD4 T-cells per condition were resuspended in 100 µL of human T-cell nucleofection solution containing 1.5 µg of Tat-expressing pcDNA3.1^(-)^ or empty vector control, and co transfected with 0.25 µg of pGFPmax plasmid using the nucleofector II device (Lonza, Basel, Switzerland) protocol U-014. Following nucleofection, cells were allowed to recover at a concentration of 2.5×10^6^ cells/ml for 4 hours in serum-free RPMI-1640 before being transferred into RPMI-20 at a concentration of 1×10^6^ cells/ml. Surface CD127 expression was monitored at 24 hour intervals by flow cytometry. GFP florescence was used as an internal control for transfection efficiency.

### Statistical analysis

All statistical analyses were performed using Microsoft Excel software. Statistical significance was measured by Students two tailed *t*-test for paired samples. Given the sample sizes of the groups, parallel nonparametric analyses were also carried out using the Wilcoxon matched pair test with 95% confidence intervals (one tailed). Statistical significance as defined by a p value greater or less than 0.05 remained the same for all comparisons irrespective of parametric or nonparametric analysis. All flow cytometry data were analysed using the FCS Express 3.0 software (De NovoSoftware, Thornhill, ON, Canada).

## Results

### Soluble HIV Tat protein down regulates surface CD127 expression on CD4 T-cells

**Figure 1:**
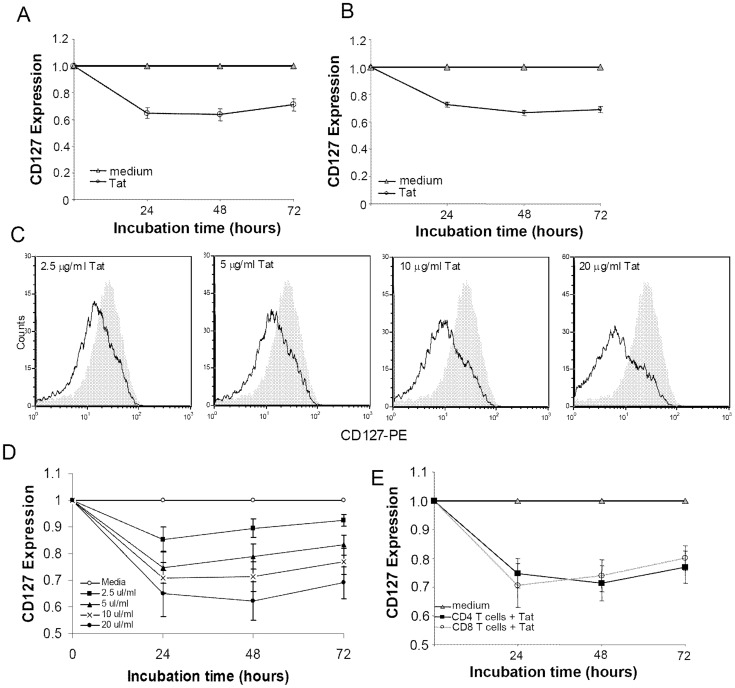
HIV-1 Tat protein down regulates CD127 surface expression on CD4 T-cells. Purified CD4 T-cells from healthy HIV-negative volunteers were incubated with Tat protein or in medium alone, stained for CD127 and analyzed by flow cytometry. (A) Percent change in CD127+ CD4 T-cells cultured with Tat (10 µg/ml) compared with media controls (n = 17; p<0.01). (B) Change in mean channel fluorescence relative to media controls for the same cultures (p<0.01). (C) Representative flow cytometry histograms from one individual showing progressive down regulation of CD127 surface expression at 48 hours with increasing amounts of Tat protein (black line) compared to medium (grey fill). (D) Composite data (n = 6; p<0.02) showing time and dose response to Tat in percent change in CD127+ CD4 T cells compared to media controls. (E) Purified CD4 and CD8 T-cells from the same healthy HIV-negative volunteers (n = 5) were incubated for up to 72 hours with HIV-1 Tat protein (10 µg/ml) or in medium alone. Percent changes in surface CD127 expression compared to medium controls are shown (p<0.01 for Tat-treated samples; p>0.5 between T-cell subsets). Graphs show mean values ± standard error of the mean (SEM).

Given the effect of soluble HIV-1 Tat protein on CD127 expression on CD8 T-cells, we wanted to determine if this HIV regulatory protein had the same effect on CD4 T-cells. To investigate this, CD4 T-cells were isolated from healthy volunteers and incubated in RPMI-20 with or without purified Tat protein (10 µg/ml) and analyzed by flow cytometry at 24 hour intervals. While CD127 surface expression varied little on cells cultured in medium alone over at least 72 hours, cells incubated in medium containing soluble Tat protein demonstrated a significant reduction in surface CD127 expression both in terms of percent positive cells and in mean channel fluorescence (Figures 1A and 1B). In the presence of exogenous Tat protein, there was a 35%±4% reduction in CD127+ CD4 T-cells relative to controls at 24 hours. This decrease leveled off at 48 hours and showed some signs of recovery by 72 hours although CD127+ cells still remained 30%±4.4% lower relative to controls.

The effect of Tat on CD127 surface expression was both time and dose dependent. Increasing amounts of Tat from 2.5 to 20 µg/ml showed a progressively greater reduction in CD127 surface expression as measured by both mean channel fluorescence (Figure 1C) and percent positive cells (Figure 1D). At 24 hours, 2.5 µg/ml Tat induced a 14.7%±4.8% decrease in CD127 expression whereas 20 µg/ml induced a 35%±8.5% reduction. Interestingly, peak suppression of CD127 occurred within 24 hours in the presence of 2.5, 5 and 10 µg/ml of Tat while peak suppression occurred at 48 hours in the presence of 20 µg/ml Tat. This may suggest Tat consumption plays a role in the recovery of CD127 at later time points.

We next asked whether soluble Tat protein reduced surface CD127 expression to the same extent comparing CD8 and CD4 T-cells. To answer this, CD8 and CD4 T-cells were purified from the same individuals (n = 5) and incubated in RPMI-20 alone or in the presence of HIV-1 Tat protein (10 µg/ml) for up to 72 hours. As seen in figure 1E, Tat down regulated CD127 expression equally on both T-cell populations. CD127 surface expression on CD4 T-cells decreased and remained within one standard deviation of that on CD8 T-cells throughout the entire incubation period (p>0.52).

We showed previously Tat dependant down regulation of CD127 on T-cells was not caused by bacterial contaminants such as LPS in the protein preparation [27]. To further test this we added *E. coli* Endotoxin (100 µg/ml CSE) to cultured CD4 T-cells and found this had no impact on CD127 surface expression (data not shown).

### HIV-1 Tat-Induced down regulation of CD127 surface expression can be blocked with heparin or anti-Tat antibodies

**Figure 2:**
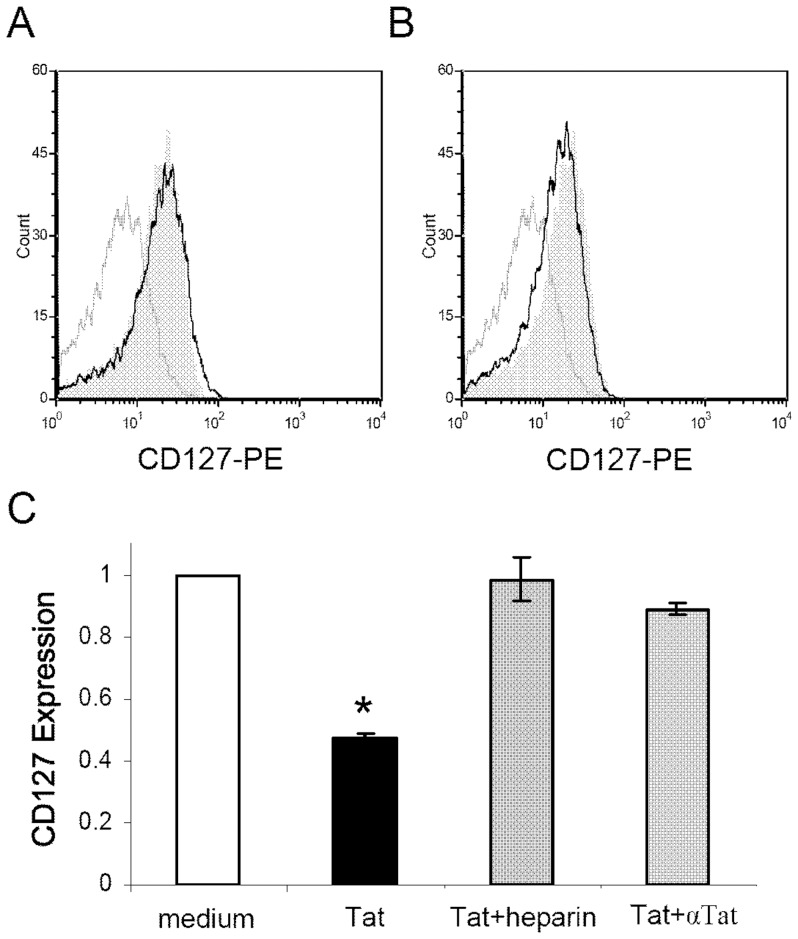
Tat-induced CD127 down regulation is blocked by heparin and anti-Tat antibodies. Purified Tat protein (2 µg) was pre-incubated with 20 USP heparin or 20 µg anti-Tat polyclonal antibodies (equimolar ratio with Tat) for 30 minutes before being added to CD4 T-cells (n = 4). Representative flow cytometry histograms show CD127 surface expression on CD4 T-cells after 24 hours incubation with (A) 10 µg/ml Tat (grey line), Tat plus heparin (black line) or in medium alone (grey fill); and (B) 10 µg/ml Tat (grey line), Tat plus anti-Tat antibodies (black line) or in medium alone (grey fill). (C) Composite data (n = 5) showing CD127 expression relative to medium controls for Tat, Tat plus heparin, and Tat plus anti-Tat antibodies after 24 hours incubation. * denotes p value <0.05 compared to media. The relative changes are mean values ± SEM.

To ensure the down regulation of surface CD127 expression on CD4 T-cells was specifically due to Tat, we pre-incubated Tat protein with either heparin or polyclonal anti-Tat antibodies to see if this would block the effect. Heparin has been shown to bind directly to Tat protein and prevent its uptake by cells from the culture medium [36], [42]. Purified Tat protein was pre-incubated for 30 minutes with an equimolar concentration of anti-Tat polyclonal antibodies (140 µg/ml) or with 100 USP/ml heparin. The Tat preparations were then added to purified CD4 T-cells and the samples were incubated for 24 hours (figure 2). Whereas cells incubated with heparin or with anti-Tat antibodies alone demonstrated no change in CD127 surface expression, 10 µg/ml Tat protein induced a 52%±7% decrease in CD127. In contrast, Tat protein pre-bound with heparin had no effect on CD127 expression (1.4%±2.1% decrease relative to media control; p = 0.61). Similarly, polyclonal anti-Tat antibodies pre-bound to Tat also inhibited Tat's ability to down regulate CD127 (11%±5.4% reduction relative to media control; p = 0.54). These data then confirm the down regulation of CD127 on the surface of CD4 T-cells is due specifically to the HIV Tat protein. The fact that anti-Tat antibodies inhibit the effect further demonstrates CD127 suppression is not due to bacterial contaminants in the Tat protein preparation.

### HIV-1 Tat-Induced down regulation of CD127 surface expression is not caused by cell death or apoptosis

**Figure 3:**
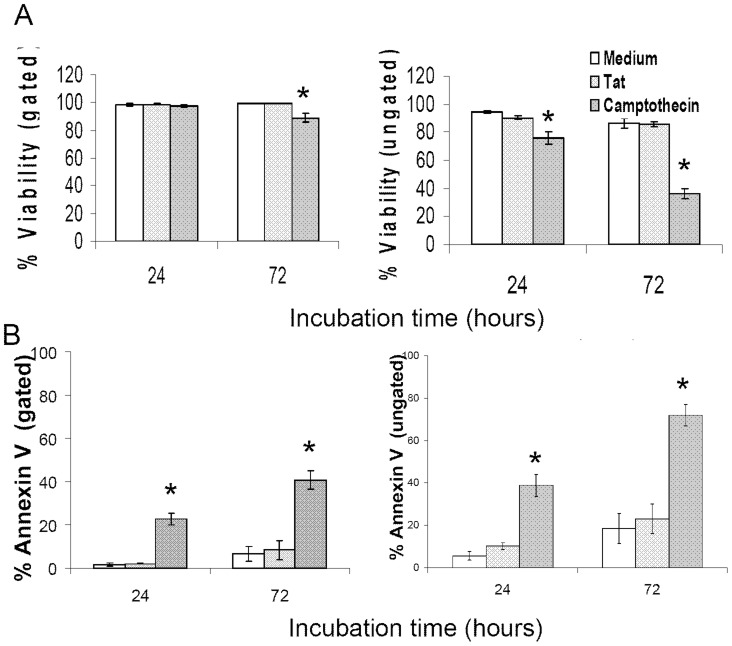
Tat protein does not affect the viability of purified CD4 T-cells. Purified CD4 T-cells from healthy HIV-negative volunteers (n = 4) were incubated in medium alone or with Tat protein (10 µg/ml) for up to 72 hours and then stained with propidium iodide or annexin V-FITC and analyzed by flow cytometry. Analyses were performed on the total ungated population as well as on only live cells based on forward and side scatter. (A) Propidium iodide exclusion showed no significant difference in viability between cells cultured in Tat or medium alone. (B) Apoptosis as indicated by positive annexin V staining averaged <10% in the gated population at 72 hours and was no different comparing cells cultured in medium alone or with Tat protein. Camptothecin (10 µM) induces apoptosis and was used as a positive control. * denotes p value <0.05 compared to media. Graphs show mean values ± SEM.

There have been conflicting reports regarding the effect of HIV-1 Tat on both promoting and inhibiting apoptosis in T-cells [43], [44], [45]. To ensure that the reduction in CD127 surface expression evident on Tat treated cells was not the result of apoptosis or cell death, purified CD4 T-cells were cultured in the presence of Tat protein (10 µg/ml) and stained with propidium iodide and annexin V at 24 and 72 hours. Cell viability as indicated by propidium iodide exclusion in the total ungated cell population was maintained at >85%±3.5% over 72 hours for cells incubated in RPMI-20 alone or in the presence of Tat protein. Within the live gate based on forward and side scatter viability was >99%±0.1% at 72 hours and again was not different comparing cells incubated in medium alone or in the presence of Tat protein (Figure 3A). Apoptosis as indicated by positive annexin V staining remained <10%±4.3% over 72 hours in the gated population regardless of the presence of Tat protein in the medium. In the ungated total population the difference in annexin V staining comparing Tat-treated cells to media control was only 4.5%, less than the standard error of 7.1% (Figure 3B). Thus, Tat-induced down regulation of CD127 surface expression is not the result of cell death or apoptosis.

### CD127 surface expression is down regulated by HIV-1 Tat protein on both naïve and memory CD4 T-cell subsets

**Figure 4:**
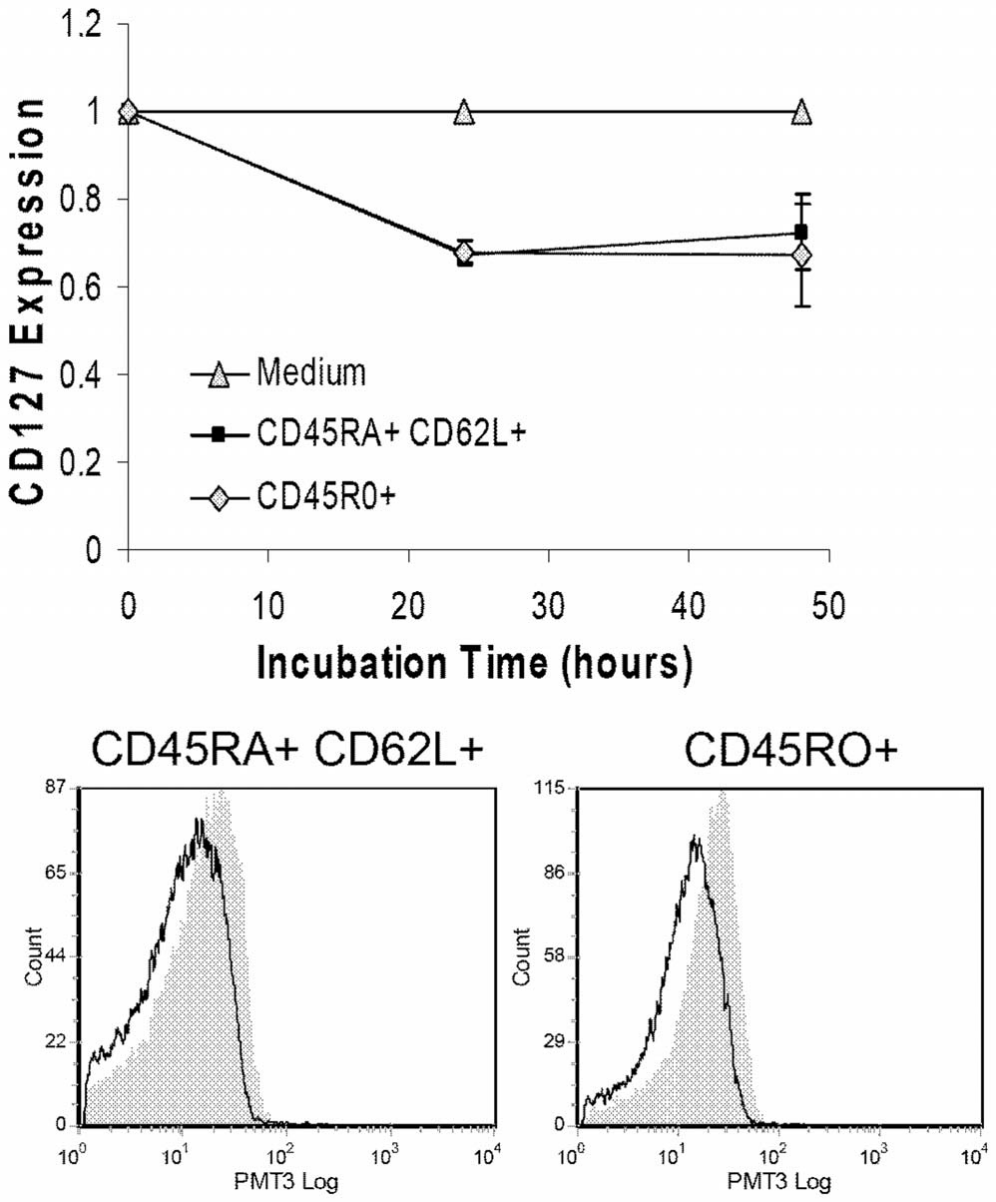
Tat down regulates CD127 surface expression equally on naïve and memory CD4 T-cell subsets. Purified CD4 T-cells from healthy HIV-negative volunteers (n = 4) were incubated in medium alone or with Tat protein (10 µg/ml) for up to 48 hours. CD127 surface expression was then measured by flow cytometry on naïve CD4 T-cells defined as CD45RA+ and CD62L+, and on memory cells defined as CD45RO+. There was little difference between the subsets (p>0.33). The changes relative to medium controls (p<0.02) are shown (mean values ± SEM). Representative histograms show CD127 expression on indicated populations at 24 hours comparing media (grey fill) to CD4 T-cells treated with Tat protein (black line).

The memory subset of CD4 T-cells has been shown to be particularly sensitive to CD127 down regulation in HIV-infected individuals [23], [46], [47]. This raised the question as to whether the HIV-1 Tat protein would have a more profound effect on this subset compared to naïve cells. Purified CD4 T-cells were incubated in RPMI-20 alone or with Tat protein (10 µg/ml) and CD127 expression was analyzed by flow cytometry gating on the different CD4 T-cell subsets. Naïve cells were defined as CD45RA^+^CD62L^+^ and memory cells were defined as CD45RO^+^. As seen in figure 4, soluble HIV Tat protein down regulated CD127 surface expression equally on naïve and memory CD4 T-cells compared to controls. Both demonstrated a 32%±2.8% decrease in CD127 at 24 hours, equivalent to what was seen in the total CD4 T-cell population.

### HIV Tat specifically down regulates CD127 and does not affect other cell surface proteins

**Figure 5:**
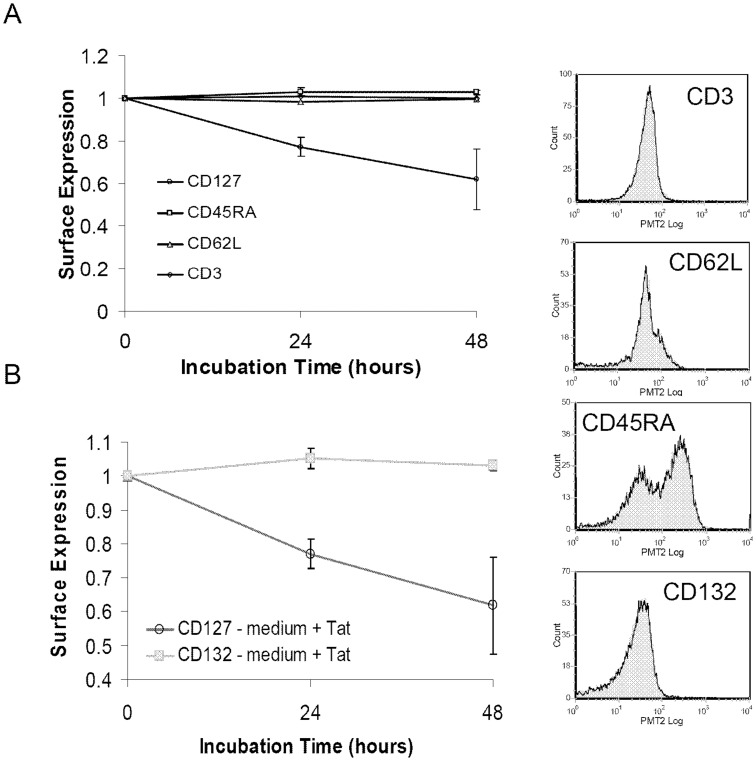
Tat does not affect other CD4 T-cell surface proteins. Purified CD4 T-cells from healthy HIV-negative volunteers (n = 4) were incubated in medium alone or with Tat protein (10 µg/ml) for up to 48 hours and then analyzed by flow cytometry. (A) Changes in positive staining for the indicated surface proteins relative to medium controls (p>0.09 for all proteins other than CD127 where p<0.04). (B) Change in CD132+ CD4 T-cells relative to medium controls (p>0.12 at 48 hours). Graph represents mean values ± SEM. Representative flow histograms show CD4 T-cells from one individual incubated in media (grey fill) or media plus Tat (black line) at 48 hours.

Internalization of soluble HIV-1 Tat protein by uninfected neighbouring CD4 T-cells occurs via clathrin-mediated endocytosis [40]. In view of this it is possible that transduction of Tat across the cell membrane could induce a non-specific and generalized down regulation of cell surface proteins. To explore this possibility, CD4 T-cells were incubated in RPMI-20 alone or with Tat protein (10 µg/ml) and then analyzed by flow cytometry for expression of a series of cell surface markers. Cells were stained for CD127, CD45RA (a marker of naïve T-cells), CD62L (a secondary lymphoid tissue homing receptor expressed on T-cells), and CD3 (an essential component of the T-cell receptor complex). Cells were also examined for the expression of CD132, the common γ-chain that associates with CD127 to form the IL-7 receptor heterodimer. Whereas Tat induced the down regulation of CD127 surface expression by 38.2%±14.2% at 48 hours relative to controls, it had no effect on the expression of the other cell surface proteins examined (Figure 5A). CD3 showed the maximum variance, only a 3.2%±2.3% difference relative to controls (Figure 5A). Similarly, Tat had no effect on the expression of CD132 (Figure 5B). After 48 hours in the presence of Tat, CD132 increased by only 3.1%±1.5% compared to controls. Thus it appears Tat specifically down regulates CD127 and does not affect at least a number of other cell surface proteins.

### CD127 down regulation by HIV-1 Tat protein is not due to CD4 T-cell activation

**Figure 6:**
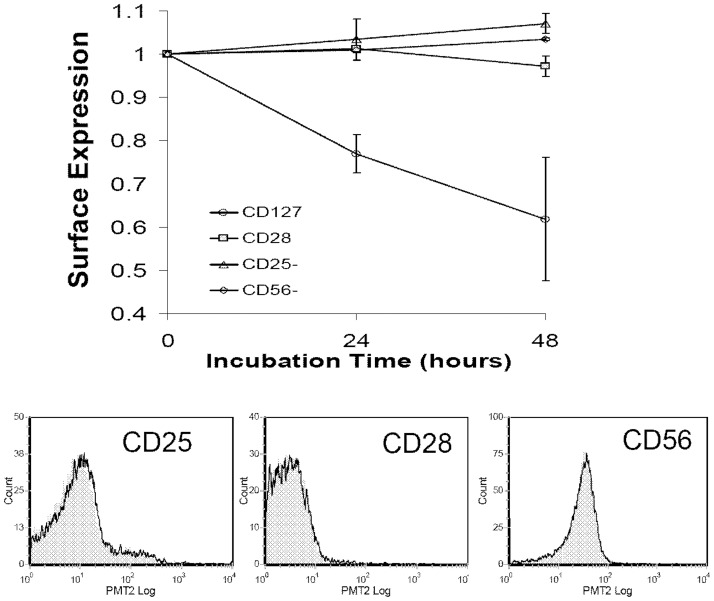
Tat-induced CD127 down regulation is not due to CD4 T-cell activation. Purified CD4 T-cells from healthy HIV-negative volunteers (n = 4) were incubated in medium alone or with Tat protein (10 µg/ml) for up to 48 hours and then analyzed by flow cytometry. The changes in surface protein expression relative to medium controls are shown (p>0.19 for all proteins other than CD127 where p<0.04 at 48 hours). Graph represents mean values ± SEM. Representative flow histograms show CD4 T-cells from one individual incubated in media (grey fill) or media plus Tat (black line) at 48 hours.

T-cell activation has been shown to down regulate CD127 surface expression on CD8 and CD4 T-cells [24], [48], [49]. Indeed, levels of CD127^-^ T-cells were shown to be directly correlated with CD8 T-cell activation [24] and in a study of 42 HIV-infected individuals receiving antiretroviral therapy, Benito et al. demonstrated CD127 down regulation was associated with activation of CD4 T-cells [49]. This raised the question as to whether Tat down regulates CD127 on the cell surface by activating CD4 T-cells. To address this question, purified CD4 T-cells were incubated in RPMI-20 alone or with Tat protein (10 µg/ml) and then examined by flow cytometry for expression of CD127 as well as the established activation markers CD25 and CD56. CD28 is constitutively expressed on naïve T-cells where it provides costimulatory signals and is down regulated upon T-cell stimulation. As seen in figure 6 and consistent with the previous data, Tat induced a significant down regulation of CD127 on the surface of CD4 T-cells. In contrast, CD28, CD25 and CD56 remained unchanged relative to controls in the presence of Tat protein. Thus Tat does not appear to induce a generalized activation of CD4 T-cells leading to the down regulation of CD127.

### Tat-induced down regulation of CD127 surface expression is reversible

**Figure 7:**
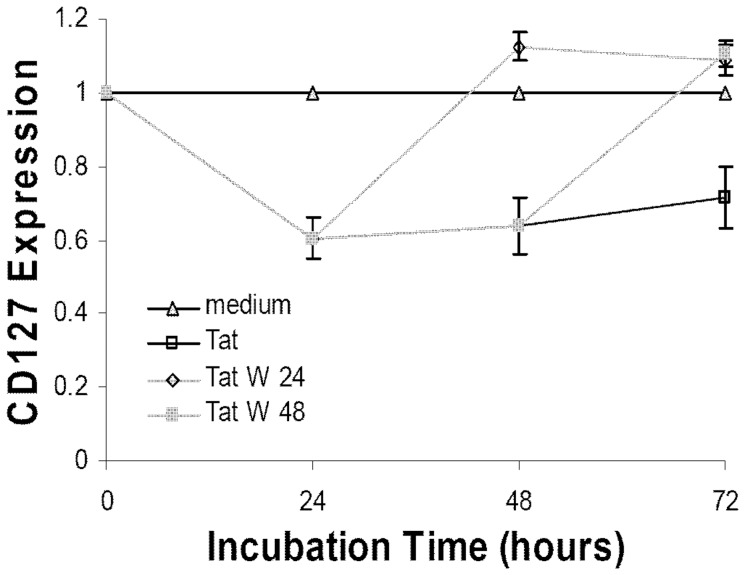
CD127 surface expression recovers on CD4 T-cells after Tat is removed from the culture medium. Purified CD4 T-cells from healthy HIV-negative volunteers (n = 4) were incubated in medium alone or with Tat protein (10 µg/ml). At 24 (Tat W 24) and 48 (Tat W 48) hours the cells were washed twice in PBS and resuspended in fresh medium in the absence of Tat protein. Cells were analyzed for CD127 surface expression by flow cytometry at the times indicated. The changes relative to medium controls are shown (mean values ± SEM).

CD127 is down regulated on CD4 T-cells in HIV infected patients and partially recovers following viral suppression with antiretroviral therapy [26]. A cohort study of patients with primary, chronic and long-term non-progressive HIV-1 infection showed that CD127 down regulation on CD4 T-cells was rapidly reversible and returned to normal levels less than 24 hours after incubation ex vivo in fresh medium [26]. To investigate whether CD127 down regulation by Tat was reversible, purified CD4 T-cells were incubated in RPMI-20 alone or with Tat protein (10 µg/ml). At 24 and 48 hours, the cells were washed and resuspended in fresh RPMI-20 without Tat. As shown in figure 7, once Tat was removed from the medium CD127 recovered on the cell surface within 24 hours. This clearly indicates Tat's effect on CD127 is reversible and that Tat is continually required to maintain suppression of CD127.

### Tat has a direct effect on CD127 expression and does not require de novo protein synthesis

**Figure 8:**
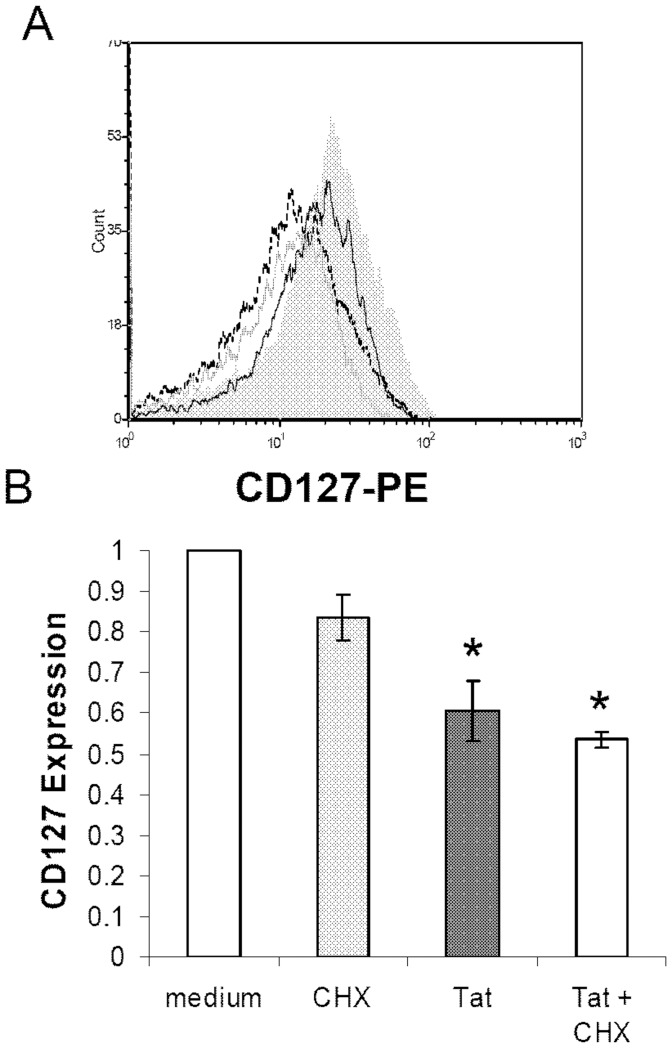
de novo protein synthesis is not required for Tat-induced down regulation of CD127. Purified CD4 T-cells from healthy HIV-negative volunteers were incubated in medium alone, with cyclohexamide (100 µM; CHX), Tat protein (10 µg/ml) or Tat plus CHX for 24 hours and then analyzed by flow cytometry. (A) Representative histogram showing CD127 surface expression on CD4 T-cells cultured in the presence of CHX alone (black line), Tat alone (dashed line) and CHX plus Tat (grey line) compared to medium control (grey fill). (B) Composite data from n = 4 demonstrating inhibition of protein synthesis with CHX does not prevent Tat-induced CD127 down regulation at the cell surface. CHX alone had no significant effect on CD127 expression (p = 0.07). There was no significant variance in surface CD127 expression between Tat and Tat + CHX treatments (p = 0.38) whereas both treatments showed significant down regulation of CD127 relative to medium (* denotes p<0.009). The percent changes relative to medium controls are shown (mean values ± SEM).

We next questioned whether soluble HIV Tat protein had a direct effect on CD127 surface expression or whether induction of a second factor was required to down regulate the receptor. To investigate this, purified CD4 T-cells were incubated in RPMI-20 alone, with Tat protein (10 µg/ml), with cyclohexamide (CHX) or with Tat protein plus CHX (figure 8). Cells treated with CHX alone represent the normal decay rate of CD127 from the cell surface in the absence of new protein synthesis. As seen in figure 8, cells incubated with CHX alone showed a 16.5%±7.3% decrease in CD127 surface expression over 24 hours. Tat protein alone induced a 39.6%±5.6% decrease in CD127. When CHX was included with Tat, there was a 46.5%±1.9% drop in CD127 surface expression indicating de novo protein synthesis is not required for Tat to have its effect.

### New protein synthesis is required for CD127 recovery following down regulation by Tat

**Figure 9:**
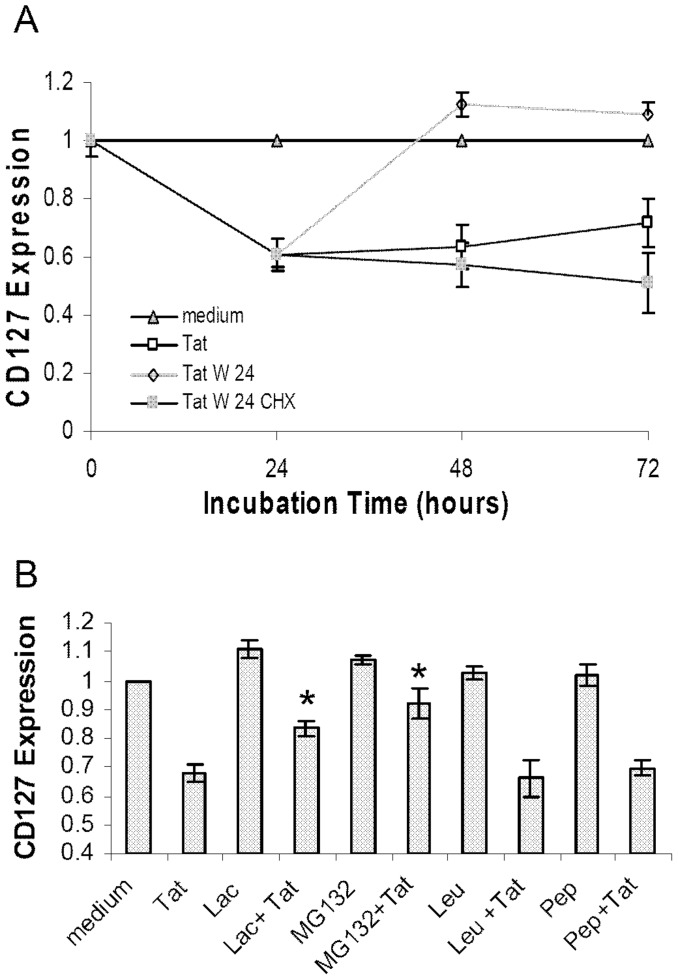
de novo protein synthesis is required for recovery of CD127 surface expression on CD4 T-cells following Tat-induced down regulation. Purified CD4 T-cells from healthy HIV-negative volunteers (n = 4) were incubated in medium alone or with Tat protein (10 µg/ml). (A) After 24 hours, cells incubated with Tat were washed twice and resuspended in fresh medium alone (Tat W 24) or in medium plus 100 µM CHX (Tat W 24 CHX). Cells were analyzed for CD127 surface expression by flow cytometry at the times indicated. (B) Cells were pre-incubated with and without proteasome inhibitors (5 µM MG132, 10 µM Lactacystin) and lysosome inhibitors (10 µM Leupeptin, 10 µg/ml Pepstatin) for 1 hour followed by incubation with 10 µg/ml Tat protein. After 24 hours, CD127 surface expression was measured by flow cytometry. The changes relative to medium controls are shown (mean values ± SEM). * denotes p value less than 0.05 compared to Tat treated control.

As shown above, CD127 recovers on the cell surface once soluble Tat protein is removed from the medium. To determine if this recovery of CD127 requires new protein synthesis, CD4 T-cells were first incubated with soluble Tat for 24 hours and then washed and re-incubated in medium alone or medium containing cycloheximide. As shown in figure 9A, cells maintained in medium containing Tat protein maintained suppression of CD127 for up to 72 hours while those washed and transferred to fresh medium alone after 24 hours recovered surface CD127 expression. However, when Tat protein was removed from the medium and the cells were then incubated in cycloheximide, CD127 failed to recover. Thus Tat induces an absolute loss of CD127 from the cell and new protein synthesis is required to replace CD127 on the cell surface. Previously we have shown HIV-1 Tat protein removes CD127 from the surface of CD8 T cells and directs it to the proteasome for degradation [41]. To determine if this is also the case in primary CD4 T cells, the cells were pre-incubated with proteasome (MG132, lactacystin) or lysosome inhibitors (leupeptin, pepstatin) for 1 hour and then incubated with HIV-1 Tat protein for 24 hours. As seen in figure 9B, Tat-induced down regulation of CD127 (33+/−3%, p value <0.01 compared to medium) was inhibited by both lactacystin and MG132, showing only a 16+/−3% (p value  = 0.02 compared to Tat treated cells) and 8+/−5% (p value compared to Tat treated cells <0.01) reduction in CD127 respectively. In contrast, cells pretreated with the lysosome inhibitors pepstatin (31+/−3 %, p value  = 0.35, compared to Tat treated cells) or leupeptin (34+/−6%, p value  = 0.41) still down regulated CD127 in response to extracelluar Tat similar to cells treated with Tat alone.

### Endogenously expressed HIV Tat protein down regulates CD127 expression

**Figure 10:**
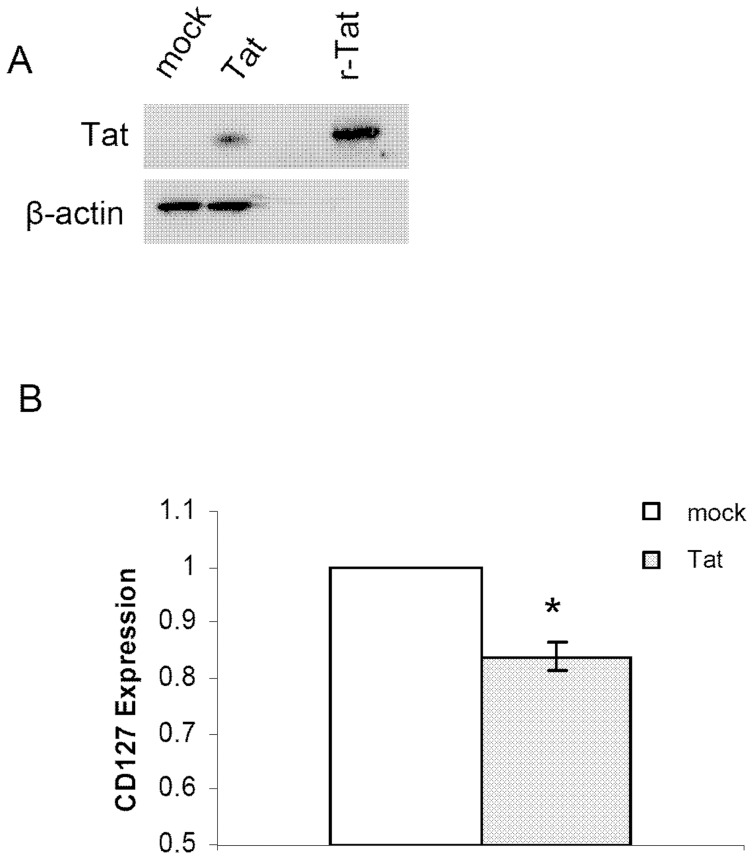
Endogenous production of Tat protein within CD4 T-cells prevents recovery of CD127 on the cell surface. Isolated CD4 T cells were stimulated with anti-CD3/anti-CD28 beads for 24 hours and then nucleofected with either pcDNA3.1^(-)^ empty vector (mock) or pcDNA3.1^(-)^ expressing Tat. (A) Tat protein expression was confirmed by western blot analysis. Cell lysates were prepared 24 hours following transfection, separated by SDS-PAGE and then analyzed by Western for Tat expression using α-Tat antibodies (clone 1102-Immunodiagnostics). Beta-actin was used as a loading control and purified recombinant Tat (r-Tat) was included to confirm the size and identity of endogenously expressed Tat protein. (B) Surface CD127 expression on CD4 T cells was measured after 24 hours by flow cytometry. Percent change in CD127 expression relative to empty pcDNA3.1^(-)^ control is shown (n = 4; mean values ± SEM). * denotes p value less than 0.05 compared to mock control.

HIV-1 Tat is secreted from infected CD4 cells and is readily internalized by uninfected bystander cells. Since less than 0.2% of peripheral CD4 T cells are productively infected with HIV [1], [2], [3], exogenous Tat protein may exert a wider influence on the CD4 T cell population. To address whether endogenously expressed Tat protein also down regulates CD127 surface expression, we expressed Tat in primary human CD4 T cells by transient transfection (Figure 10A). This was achieved by first stimulating the cells with anti-CD3/anti-CD28 beads for 24 hours followed by transfection with the pcDNA3.1^(-)^ empty vector or pcDNA3.1^(-)^ expressing Tat. CD127 expression was then measured by flow cytometry at 24 hours. As seen in figure 10B, CD4 T cells transfected with the Tat expressing plasmid had lower CD127 expression (18+/−3, p value  = 0.01) compared to CD4 T cells transfected with the empty vector.

### HIV-1 Tat protein inhibits IL-7-induced up regulation of Bcl-2

**Figure 11:**
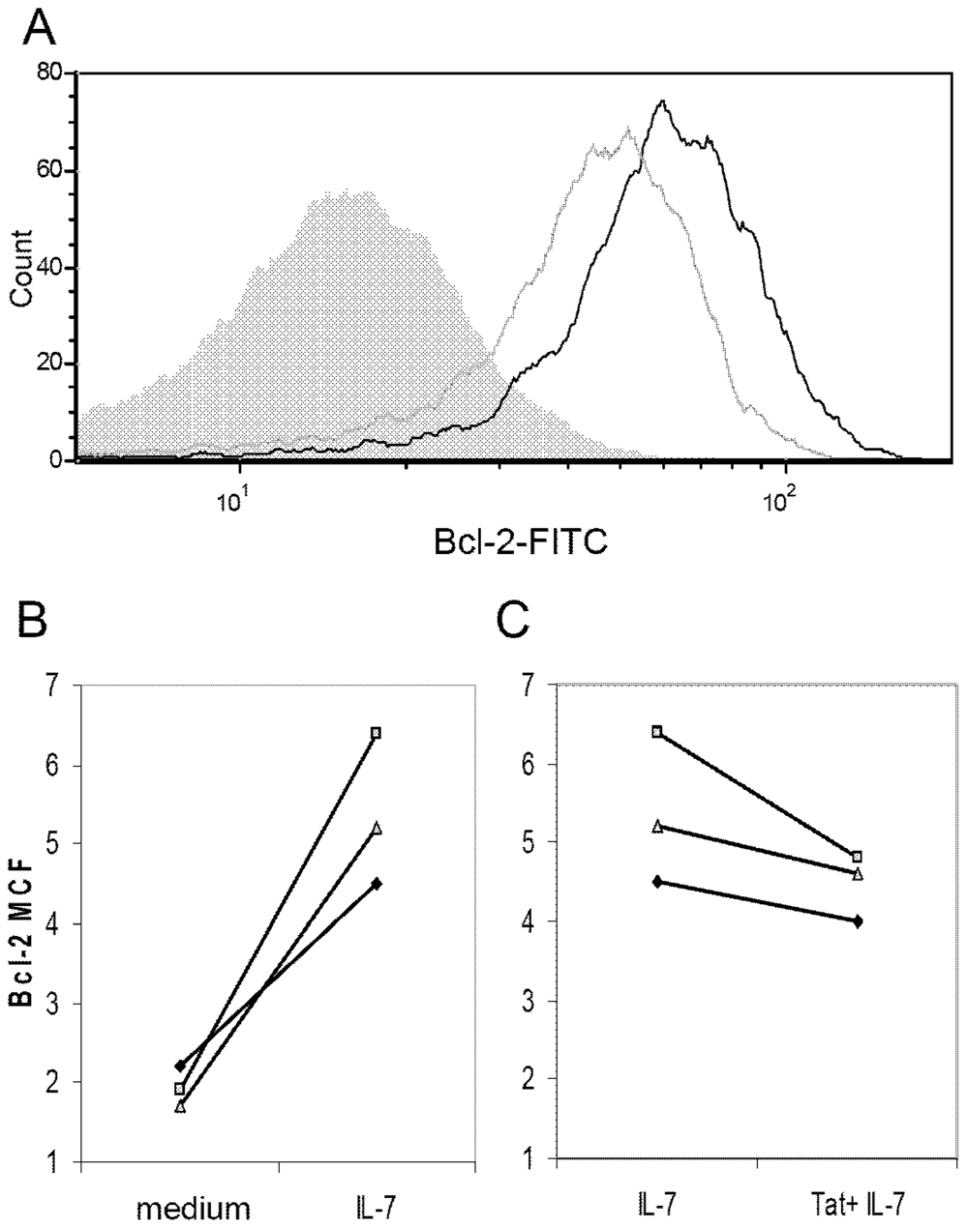
Soluble HIV-1 Tat protein inhibits IL-7 induced Bcl-2 expression in CD4 T-cells. Purified CD4 T-cells from healthy HIV-negative volunteers were pre-incubated in medium alone or in medium containing Tat protein (10 µg/ml) for 48 hours. Cells were then stimulated with IL-7 (10 ng/ml) for an additional 72 hours and then fixed, stained for Bcl-2 expression and analyzed by flow cytometry. (A) Representative flow cytometry histogram showing Bcl-2 expression in untreated CD4 T-cells (grey fill), following IL-7 stimulation (black line), and following IL-7 stimulation of Tat pre-treated cells (grey line). (B, C) Bcl-2 expression as measured by mean channel fluorescence (MCF) in (B) CD4 T cells treated with IL-7 compared to medium controls, and (C) cells pre-incubated in medium or with Tat prior to IL-7 stimulation (n = 5).

IL-7 signaling plays an essential role in CD4 T-cell proliferation and homeostasis and has been specifically shown to up regulate expression of the anti-apoptotic molecule B cell leukemia/lymphoma 2 (Bcl-2) [50], [51]. Because Tat protein down regulates the IL-7 receptor alpha-chain on the surface of CD4 T-cells, we wanted to determine whether this effect was functionally significant. To investigate this, purified CD4 T cells were pre-incubated in RPMI-20 alone or with Tat protein (10 µg/ml) for 48 hours and then stimulated with IL-7 (10 ng/ml). While IL-7 as expected induced the expression of Bcl-2, pre-incubation with Tat protein significantly blunted IL-7's effect. The cells pre-incubated with Tat protein before administration of IL-7 showed diminished up regulation of Bcl-2 as demonstrated by mean channel fluorescence of Bcl-2 positive stained cells. The maximum effect is a 25% decrease in the MCF from cells treated with IL-7 alone compared to cells pretreated with Tat protein followed by IL-7 that was statistically significant (pvalue  = 0.04) (Figure 11).

## Discussion

HIV-1 influences T-cell function and homeostasis throughout the course of infection leading to quantitative effects within the CD4 T-cell population and qualitative effects in both CD4 and CD8 T cells. Homeostatic maintenance within the CD4 T cell population is largely dependent upon IL-7 signaling. IL-7 prolongs T cell survival through induction of the anti-apoptotic factors Bcl-2 and Bcl-xL as well as inhibition of the pro-apoptotic factors Bad and Bax [15]. IL-7, in combination with IL-15, has also been shown to initiate proliferation in human memory CD4 T cells and its absence results in substantial depletion and lack of regeneration in this lymphocyte population [18]. IL-7 is also important in T-cell development [52] and initiation of cellular responses [53], [54].

IL-7 signaling is dependent upon heterodimerization of the IL-7 receptor complex consisting of the subunits CD132 and CD127 [55]. Down regulation of CD127 surface expression on CD4 and CD8 T-cells is associated with HIV infection [22], [24], [26], [27]. Viremia results in increased plasma IL-7 levels [56], [57] which are inversely correlated with CD4 T-cell counts during HIV infection [9]. The decreased expression of CD127 on CD4 and CD8 T-cells in HIV+ patients results in poor responses to IL-7 including decreased induction of anti-apoptotic factors [28] and CD25 expression [58]. Reduced CD127 expression and subsequent impaired IL-7 signaling would be expected to have significant effects on T-cell homeostasis and function.

Tat is secreted by infected CD4+ cells and can affect the function of uninfected neighboring bystander cells [32]. We have shown previously that soluble Tat protein specifically down regulates CD127 expression on the surface of CD8 T-cells. Tat is initially internalized by CD8 T cells via endocytosis, exits the endosome through a process dependent upon the usual acidification of these vesicles, and then localizes to the inner leaflet of the cell membrane where it associates with the cytoplasmic tail of CD127 inducing receptor clustering, internalization and subsequent proteosomal degradation [41]. This loss of CD127 expression leads to inhibition of CD8 T-cell proliferation and perforin synthesis which normally follow IL-7 stimulation.

We show here that the HIV Tat protein also down regulates CD127 on the surface of CD4 T cells. This in addition to its effect on CD8 T cells expands the range of influence for this soluble accessory viral protein. Tat's effect on CD127 surface expression is similar comparing CD4 and CD8 T-cells, is dose- and time-dependent, is reversible, and is not the result of nonspecific T cell activation, necrosis or apoptosis. Furthermore, Tat selectively down regulates CD127 leaving the common ϒ-chain (CD132) and a number of other surface markers unaffected. We also demonstrated the specificity of Tat's effect on CD127 with both heparin and anti-Tat antibodies. These experiments further indicate the effect is not due to bacterial contaminates in the Tat protein preparation. Finally we also showed Tat dependant down regulation of CD127 has a functional impact on CD4 T cells, and compromises IL-7 dependant up regulation of Bcl-2. The very similar effect of Tat on CD127 expression in both CD4 and CD8 T cells suggests a likely common mechanism. Whether this mechanism operates in other lymphocyte populations expressing the IL-7 receptor is a matter of speculation. However, the fact that Tat has been shown to have the opposite effect and up regulates CD127 on monocyte-derived macrophages [60] suggests this viral protein may exert it effect through cell-specific signaling pathways.

CD4 T cells are infected by HIV and we show here endogenously produced Tat protein also down regulates CD127 expression. However, since only 0.2% of CD4 T cells in the peripheral circulation are infected, release of Tat from infected cells and the ability of soluble Tat to down regulate CD127 on bystander cells extends the influence of this HIV accessory protein on the CD4 T cell population. Down regulation of CD127 and the resultant decrease in IL-7 signaling in turn leads to reduced expression of the anti-apoptotic protein Bcl-2. Through this mechanism Tat is able to disrupt CD4 T cell homeostasis. Indeed, recent studies have shown that naïve CD4 T-cells that are not productively infected are destroyed by innate immune cells through type 1 interferon activation of caspase-1 [7], [8]. By this process, abortive infection causes accumulation of incomplete HIV DNA transcripts, leading to interferon type 1 activation of caspase-1 resulting in cell death by pyroptosis, a highly inflammatory form of apoptosis [7], [8]. It is possible that lower expression of CD127 induced at least in part by HIV-1 Tat protein contributes to this destructive process by making cells more susceptible to cell death through down regulation of Bcl-2.

While free Tat protein has been measured in the blood plasma of HIV-infected individuals at concentrations ranging 4–550 nM [33], [59], we have used considerably higher concentrations (10 µg/mL or 670 µM) in our in vitro experiments. Considerable caution, however, should be exercised when comparing in vivo fluids to in vitro assays. First, the Tat protein used in our experiments is recombinant protein produced in *E coli* and as such is not post-translationally modified. Tat is acetylated in eukaryotic cells at Lys28 and Lys50 and these modifications have been shown to enhance Tat activity in transcription assays [60], [61]. Further, protein produced in *E coli* and purified over columns is unlikely to retain 100% biological activity. Clearly, in vivo concentrations of post-translationally modified Tat secreted from neighboring HIV-infected CD4 cells and in vitro concentrations of recombinant Tat protein purified in the laboratory from *E. coli* may not be comparable. In addition to these considerations, the micro-environment in vivo is distinct from that in vitro. Indeed, the concentration of Tat in primary and secondary lymphoid tissue is unknown but given lymphoid tissues contain the highest concentration of actively infected CD4 cells [62], [63], [64], [65], it is likely the concentration of extracellular Tat in the lymphoid microenvironment is considerably higher than that of peripheral blood. In addition, Tat is not only trapped by cell surface glycans in the extracellular matrix [66] but is also rapidly taken up by neighboring cells in blood and lymphoid tissue. Thus the amount of free Tat measured in blood plasma may be only a fraction of the Tat protein available to cells in vivo. Finally, cell-cell interactions in vivo that allow efficient transfer of Tat protein from HIV-infected to uninfected cells are not likely to be efficiently recapitulated in vitro.

Therapeutic advantages have been explored in regards to HIV-1 Tat. In fact, a phase I clinical trial using a Tat variant as a vaccine showed safe induction of a balanced T_h_1/T_h_2 immune response and anti-Tat antibody production [67]. A Phase II trial has further shown that anti-Tat vaccination increased the viability and immune function of T cells [68]. This could have occurred in part by blocking the effect that Tat has on CD127 expression. Indeed, in the present study we have shown that exogenous Tat protein decreased the IL-7 induced induction of the anti-apoptotic protein Bcl-2 (Figure 11). Thus by blocking Tat, a therapeutic anti-Tat vaccination may have been able to restore at least to some extent T cell function and homeostasis. Our data support ongoing research in this area and the further development and investigation of anti-Tat vaccines.
